# Supplementary material for: Ecophylogeny of the endospheric root fungal microbiome of co-occurring Agrostis stolonifera
Source: PeerJ. 2017 Jun 8;5:e3454. doi: 10.7717/peerj.3454 (PMC5466812; doi:10.7717/peerj.3454)
Supplement: Supplemental Information 1 — Figure S1. Mean rarefaction curves of 19 samples. Coloured area (i.e., RNA fraction in red, DNA fraction in blue) indicates ±SE. Figure S2. Taxonomic distribution of sequences in the DNA and the RNA fractions. The mean relative sequence abundance and standard error are shown for the dominating groups of fungi forming the root fungal microbiota. Figure S3. Effect of sampling effort on the core microbiome size. Data were randomly resampled 1,000 times for each sampling size to calculate the mean core microbiome size and standard deviation (i.e., dashed grey and black lines for DNA and RNA fractions respectively). Figure S4. Phylogenetic tree of the Glomeromycota related root fungal microbiome OTUs. ML tree based on 432 bp of SSU rRNA gene sequences amplified from roots of Agrostis stolonifera. The tree was constructed using representative sequences of the OTUs (taxa without names) and the closest reference sequences (taxa names in italic) from the non-redundant SILVA SSURef ARB database (release 115). Barplots represent the mean expression ratio for each OTU among all samples. Null values indicate that this OTU was not detected in the RNA fraction, value = 1 indicates that this OTU was not detected in the DNA fraction, value = 0.5 indicates that the sum of the relative abundance between DNA and RNA fractions was equal. Green bars: values below 0.5, red bars: values ≥0.5. Error bars indicate ±SE. Grey circles indicate the relative abundance of each OTU in the whole dataset. Node support values above 50 are given in the following order: bootstrap values and Bayesian posterior probabilities. Figure S5. Phylogenetic tree of the Ascomycota related root fungal microbiome OTUs. ML tree based on 432 bp of SSU rRNA gene sequences amplified from roots of Agrostis stolonifera. The tree was constructed using representative sequences of the OTUs (taxa without names) and the closest reference sequences (taxa names in italic) from the non-redundant SILVA SSURef ARB database (release [file peerj-05-3454-s001.doc]

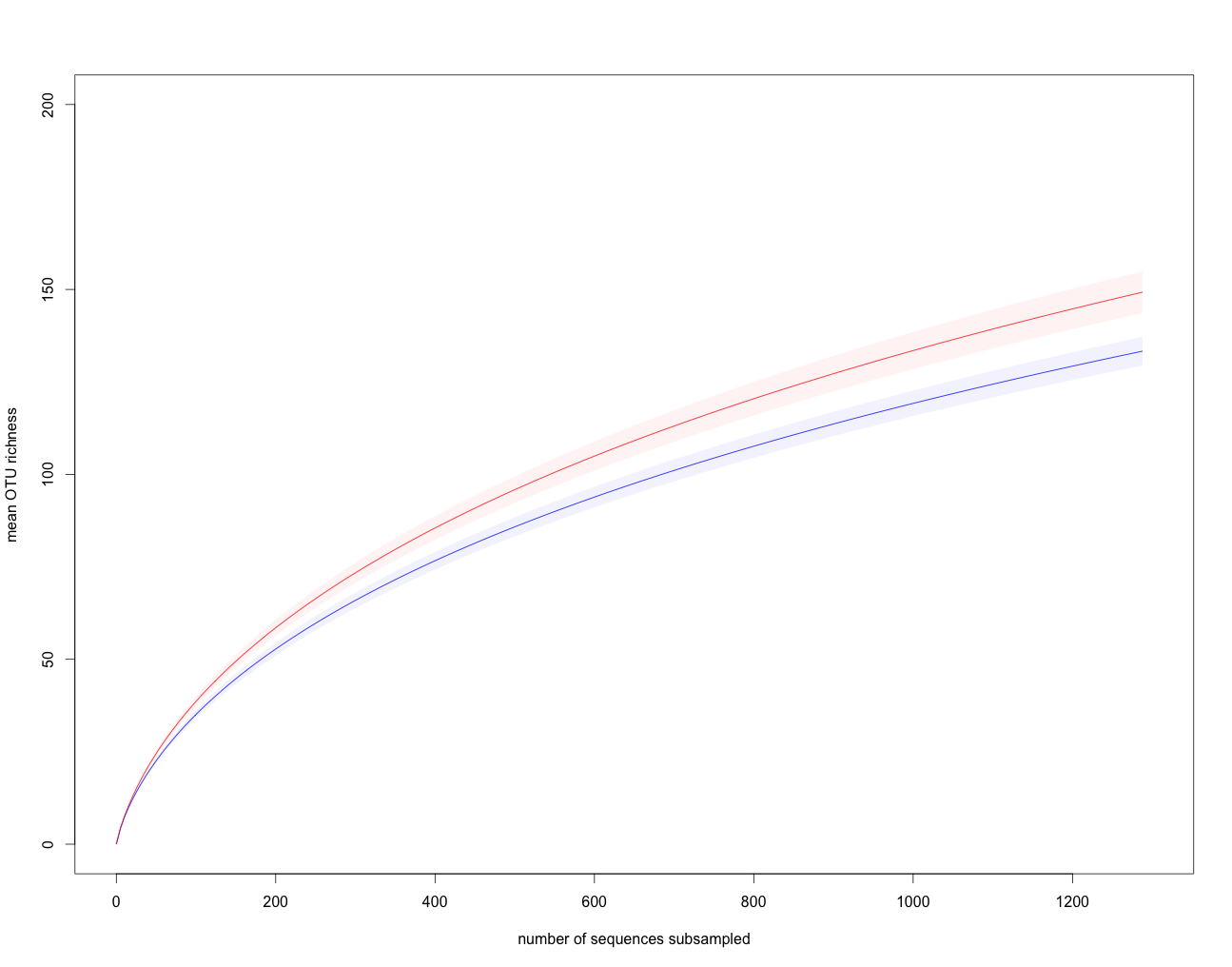
**Figure S1.** Mean rarefaction curves of 19 samples. Coloured area (i.e. RNA fraction in red, DNA fraction in blue) indicates +- SE.


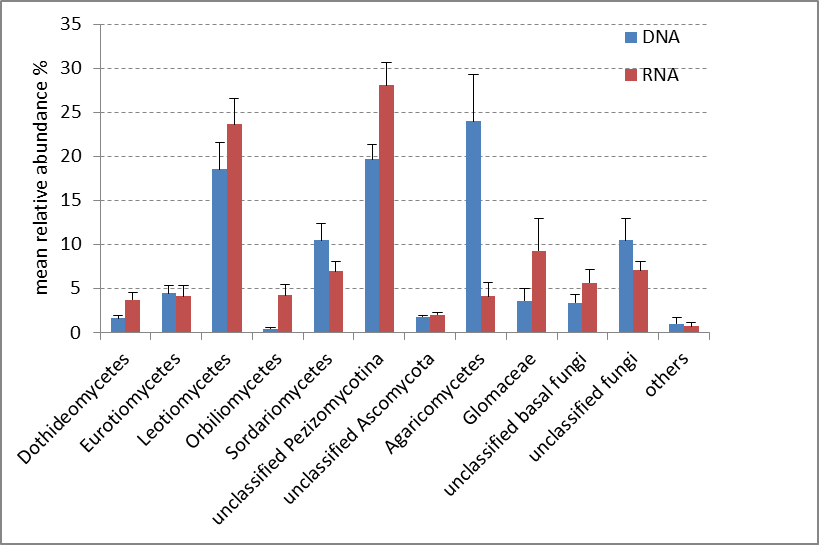


**Figure S2.** Taxonomic distribution of sequences in the DNA and the RNA fractions. The mean relative sequence abundance and standard error are shown for the dominating groups of fungi forming the root fungal microbiota.


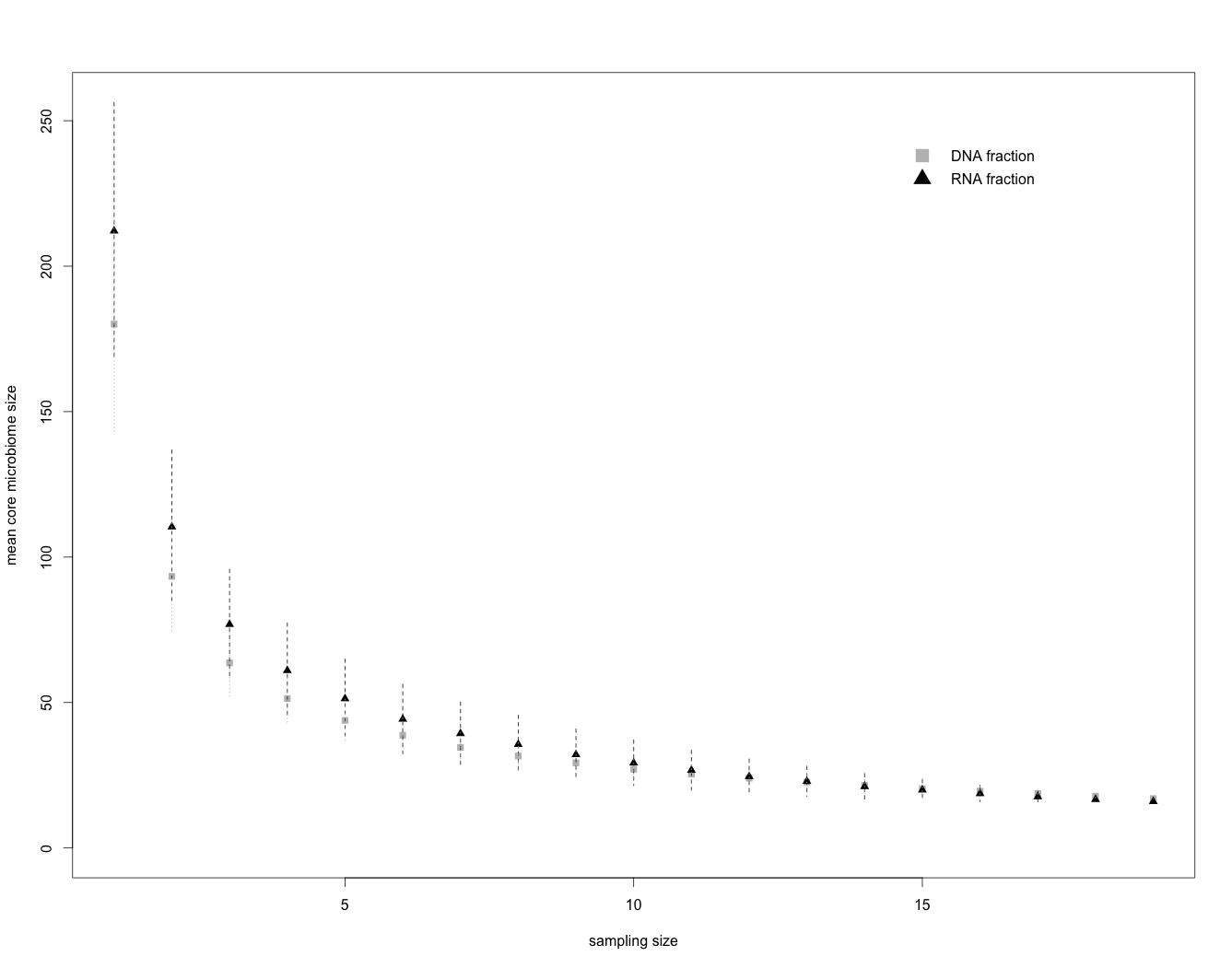
**Figure S3.** Effect of sampling effort on the core microbiome size. Data were randomly resampled 1000 times for each sampling size to calculate the mean core microbiome size and standard deviation (i.e. dashed grey and black lines for DNA and RNA fractions respectively).


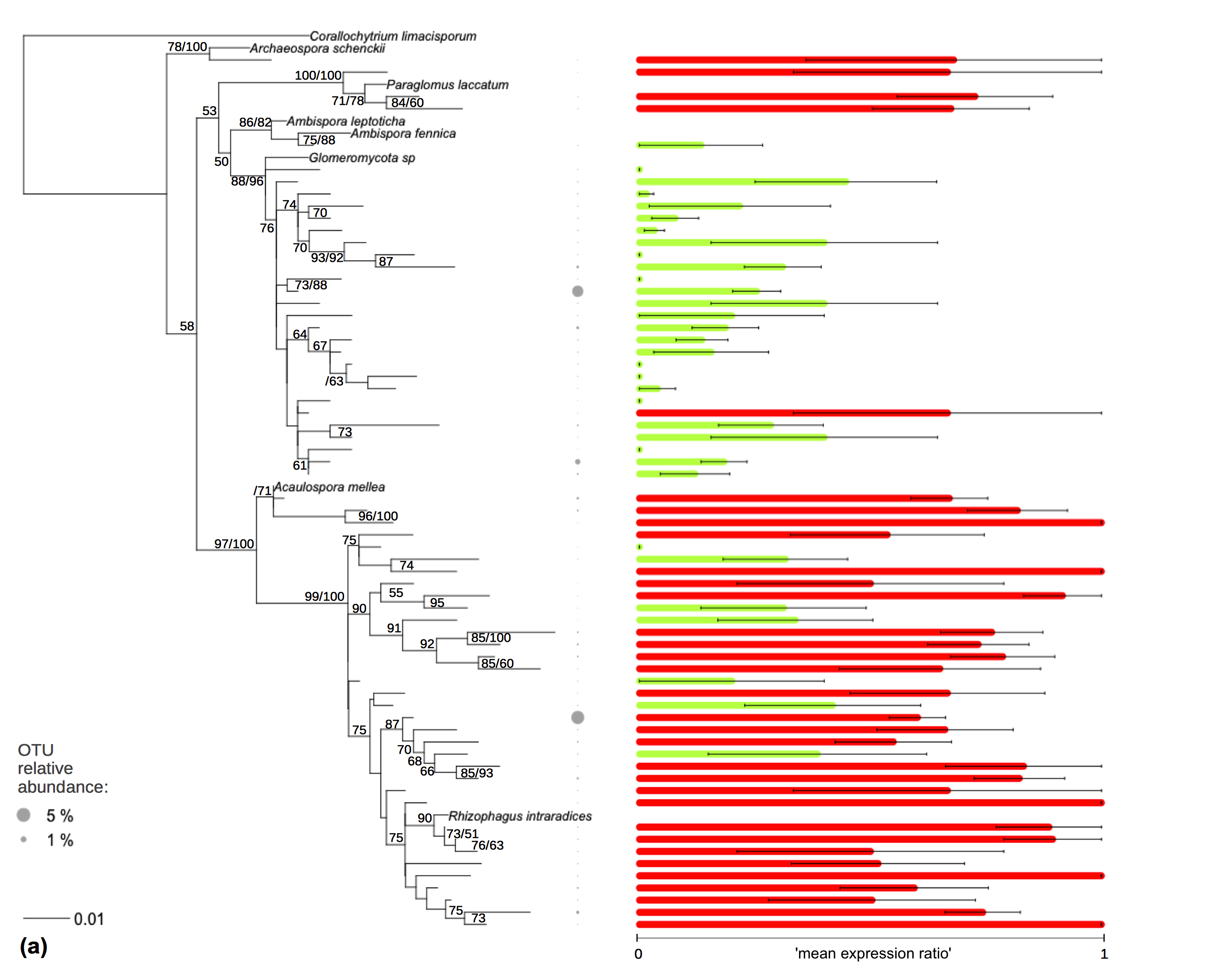


**Figure S4.** Phylogenetic tree of the Glomeromycota related root fungal microbiome OTUs. ML tree based on 432 bp of SSU rRNA gene sequences amplified from roots of *Agrostis stolonifera*. The tree was constructed using representative sequences of the OTUs (taxa without names) and the closest reference sequences (taxa names in italic) from the non-redundant SILVA SSURef ARB database (release 115).  Barplots represent the mean expression ratio for each OTU among all samples. Null values indicate that this OTU was not detected in the RNA fraction, value = 1 indicates that this OTU was not detected in the DNA fraction, value = 0.5 indicates that the sum of the relative abundance between DNA and RNA fractions was equal. Green bars: values below 0.5, red bars: values ≥ 0.5. Error bars indicate ± SE. Grey circles indicate the relative abundance of each OTU in the whole dataset. Node support values above 50 are given in the following order: bootstrap values and Bayesian posterior probabilities.

Figure S5. *Phylogenetic tree of the Ascomycota related root fungal microbiome OTUs. ML* tree based on 432 bp of SSU rRNA gene sequences amplified from roots of Agrostis stolonifera. The tree was constructed using representative sequences of the OTUs (taxa without names) and the closest reference sequences (taxa names in italic) from the non-redundant SILVA SSURef ARB database (release 115).Barplots represent the mean expression ratio for each OTU among all samples. Null values indicate
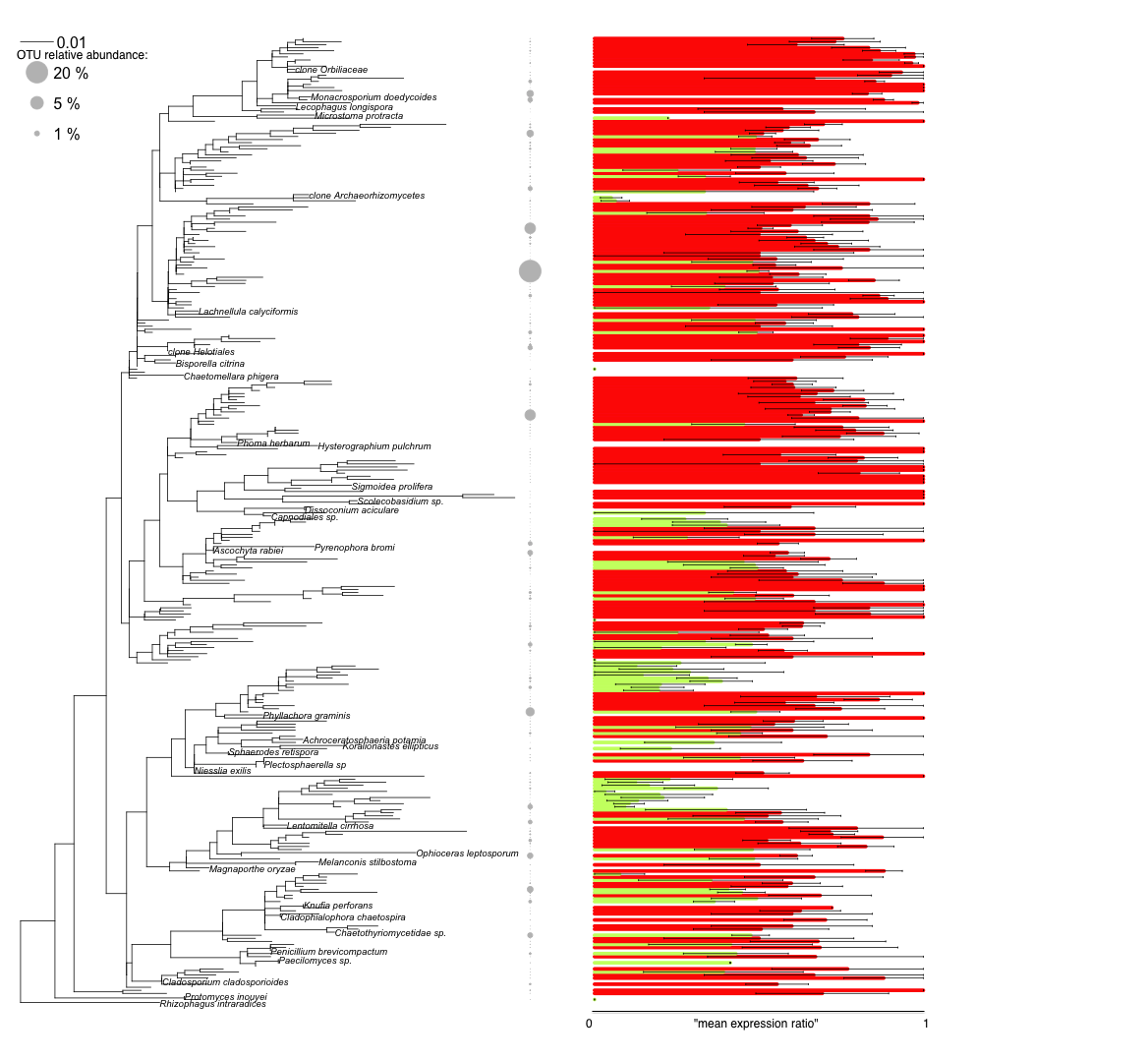
absence of this OTU in RNA fraction, value = 1 indicates that this OTU was not detected in DNA fraction, value = 0.5 indicates that the sum of the relative abundance is equal between DNA and RNA fractions. Green bars: values below 0.5, red bars: values ≥ 0.5. Error bars indicate ± SE. Grey circles indicate the relative abundance of each OTU in the whole dataset.

**Table S1.** Sequences of the fusion primers.

| **Primer Name** | **454 adaptor** | **MID** | **specific primer** |
| --- | --- | --- | --- |
| SSU-0817 | 5'-CCATCTCATCCCTGCGTGTCTCCGACTCAG | 6 to 10 N | TTAGCATGGAATAATRRAATAGGA-3' |
| NS22B | 5'-CCTATCCCCTGTGTGCCTTGGCAGTCTCAG | - | AATTAAGCAGACAAATCACT-3' |
